# Energetic performance analysis of staged palliative surgery in tricuspid atresia using vector flow mapping

**DOI:** 10.1186/s12947-017-0118-3

**Published:** 2017-12-14

**Authors:** Mao Kinoshita, Koichi Akiyama, Keiichi Itatani, Ayahiro Yamashita, Maki Ishii, Atsushi Kainuma, Yoshinobu Maeda, Takako Miyazaki, Masaaki Yamagishi, Teiji Sawa

**Affiliations:** 1 Department of Anesthesiology, Kyoto Prefectural University of Medicine, 465 Kajii Cho, Hirokoji Agaru, Kawaramachi Street, Kamigyo Ward, Kyoto City, Kyoto Prefecture 602-8566 Japan; 2 Department of Cardiovascular Surgery, Kyoto Prefectural University of Medicine, Kyoto, Japan

**Keywords:** Palliative surgery, Blalock-Taussig shunt, Bidirectional cavopulmonary shunt, Single ventricle, Tricuspid atresia, Vector flow mapping

## Abstract

**Background:**

Staged palliative surgery markedly shifts the balance of volume load on a single ventricle and pulmonary vascular bed. Blalock-Taussig shunt necessitates a single ventricle eject blood to both the systemic and pulmonary circulation. On the contrary, bidirectional cavopulmonary shunt release the single ventricle from pulmonary circulation.

**Case presentation:**

We report a case of tricuspid atresia patient who underwent first palliative surgery and second palliative surgery. Volume loading condition was assessed by energetic parameters (energy loss, kinetic energy) intraoperatively using vector flow mapping. These energetic parameters can simply indicate the volume loading condition.

**Conclusion:**

Vector flow mapping was useful tool for monitoring volume loading condition in congenital heart disease surgery.

**Electronic supplementary material:**

The online version of this article (10.1186/s12947-017-0118-3) contains supplementary material, which is available to authorized users.

## Background

There are many types of congenital heart disease collectively referred to as functionally univentricular heart. Patients with a functionally univentricular heart are recommended staged palliative surgery. The first palliative surgery is the Blalock–Taussig shunt (BTS) or pulmonary artery banding, which regulates the pulmonary blood flow. The second palliative surgery is the bidirectional cavopulmonary shunt (BCPS), which is usually performed at the age of around 6 months when pulmonary vascular resistance has sufficiently decreased after birth, but it can also be performed as early as at the age of 2 months [1, 2]. The final palliative surgery is a total cavopulmonary connection, which is usually performed between the age of 2 and 4 years when the pulmonary vascular bed has sufficiently grown. We present a case of staged palliative surgery due to tricuspid atresia in which energetic performance was analyzed using vector flow mapping.

## Case report

**Fig. 1:**
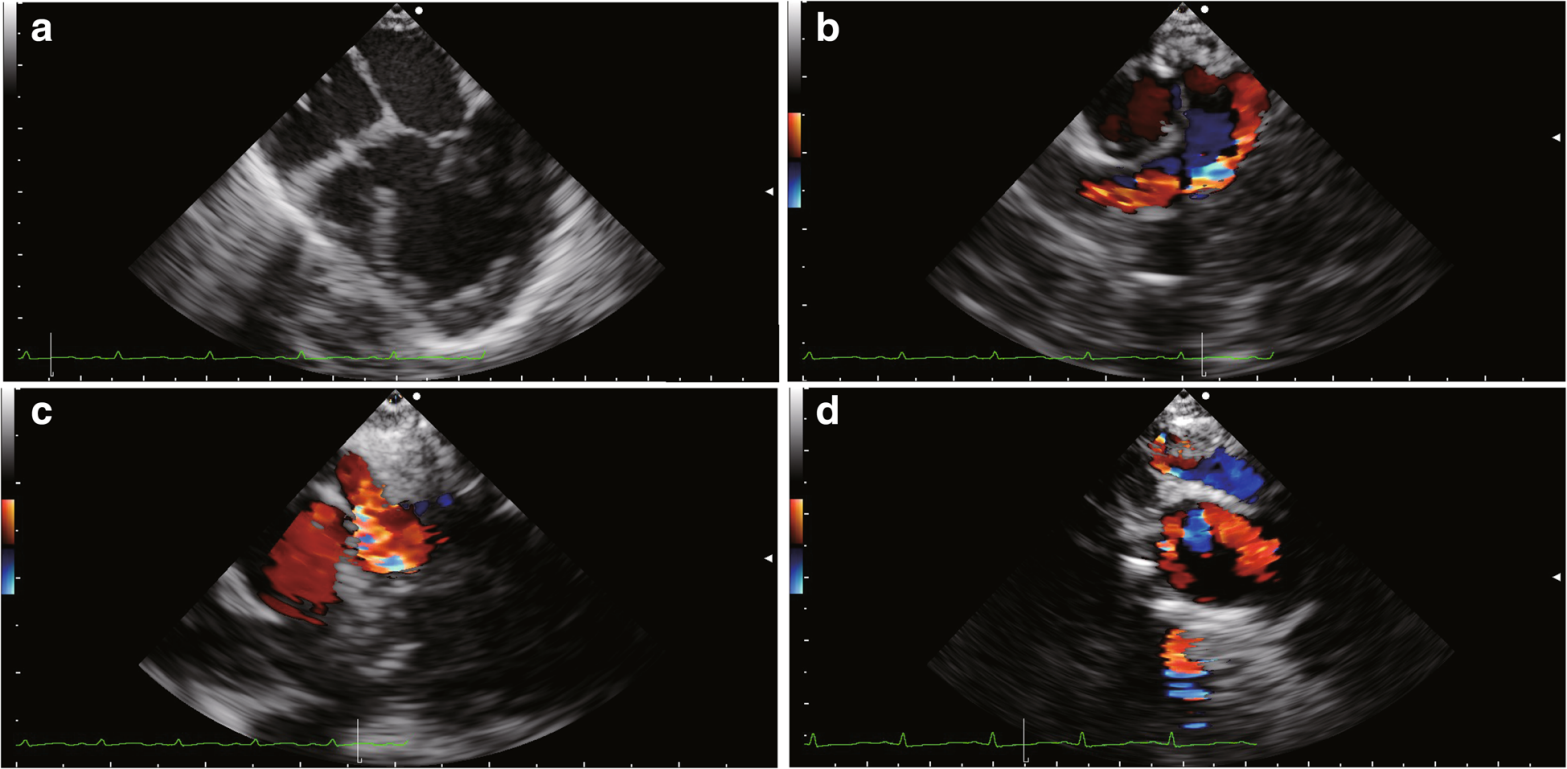
Intraoperative transesophageal echocardiographic images. **a** Midesophageal four-chamber view demonstrating tricuspid atresia, a hypoplastic right ventricle, and VSD. **b** Midesophageal right ventricle inflow–outflow view using color Doppler imaging demonstrating pulmonary stenosis that caused a dissipative flow in the main pulmonary artery before BTS. **c** Midesophageal ascending aortic short-axis view using color Doppler imaging demonstrating an antegrade flow from the completely severed main pulmonary artery to both the right and left pulmonary arteries due to BTS, which connected the brachiocepharic artery to the main pulmonary artery. **d**, Midesophageal ascending aortic short-axis view demonstrating the retrograde flow from the right pulmonary artery to the left pulmonary artery due to the BCPS, which connected the superior vena cava to the right pulmonary artery

**Fig. 2:**
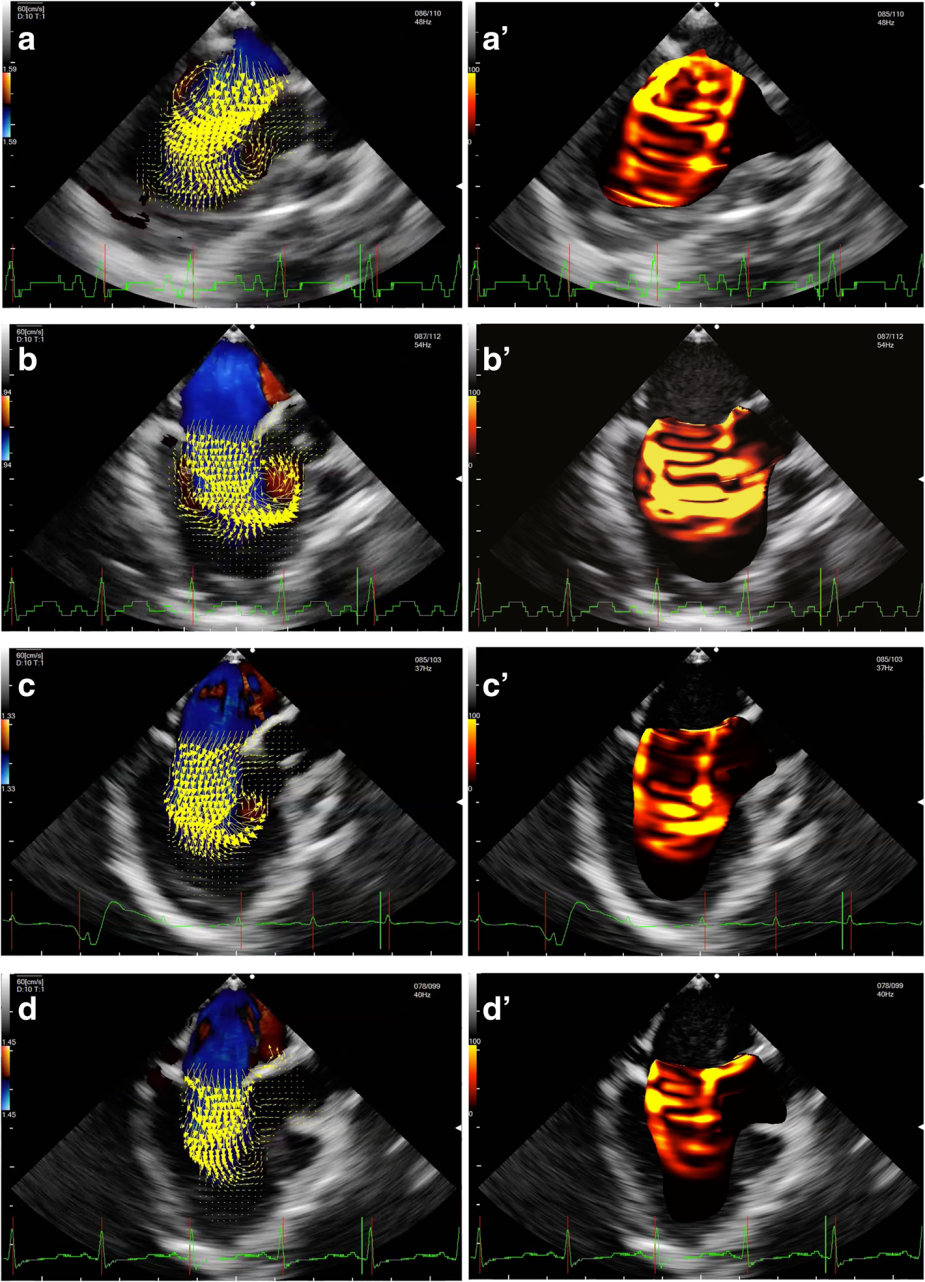
Vector flow mapping and energy loss images in midesophageal long-axis view. Brightness indicates energy loss. **A**, Vector flow mapping image before BTS. **A’**, Energy loss image before BTS. **B**, Vector flow mapping image after BTS. **B′**, Energy loss image after BTS. The large bright area indicates increased energy loss compared to Fig. **A’** due to the hyperdynamic state. **C**, Vector flow mapping image before BCPS. Volume loads decrease compared to Fig. **B** due to the worsened pulmonary blood flow. **C′**, Energy loss image before BCPS. **D**, Vector flow mapping image after BCPS. **D’**, Energy loss image after BCPS. The small bright area indicates decreased energy loss compared to Fig. **C′** due to the hypodynamic state

**Fig. 3:**
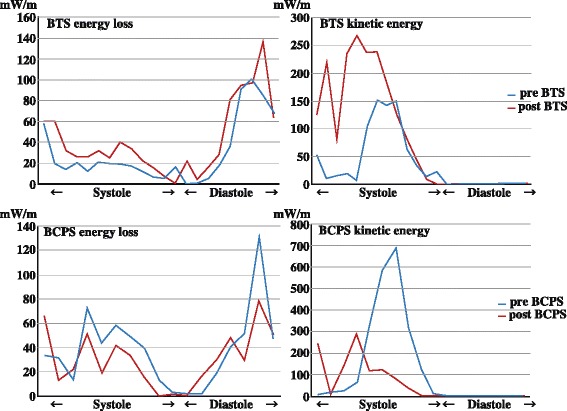
Energy loss and kinetic energy graph during one cardiac cycle. **Left upper panel**, Energy loss graph before and after BTS. Energy loss increased after BTS. **Right upper panel**, Kinetic energy graph of the left ventricular outflow tract before and after BTS. Kinetic energy increased after BTS. **Left lower panel**, Energy loss graph before and after BCPS. Energy loss decreased after BCPS. **Right lower panel**, Kinetic energy graph of the left ventricular outflow tract before and after BCPS. Kinetic energy decreased after BCPS

A male infant born at 38 weeks of gestation was diagnosed with tricuspid atresia and hypoplastic right ventricle by a fetal ultrasound scan. Transthoracic echocardiography revealed tricuspid atresia, ventricular septal defect (VSD), pulmonary stenosis, patent ductus arteriosus (PDA), and patent foramen ovale immediately after birth. E-type prostaglandin was prescribed to prevent spontaneous closure of PDA. However, this closure could not be prevented and his oxygen saturation level dropped to 75%. He then underwent BTS and main pulmonary artery division for first palliation at the age of 2 months. A midesophageal four-chamber view using transesophageal echocardiography (TEE) revealed tricuspid atresia, a hypoplastic right ventricle, and VSD (Fig. 1a; Additional files 1 and 2: Video Clips 1 and 2). A midesophageal right ventricle inflow–outflow view using color Doppler imaging demonstrated pulmonary stenosis that caused dissipative flow in the main pulmonary artery before the procedure (Fig. 1b; Additional file 3: Video Clip 3). After the procedure, a midesophageal ascending aortic short-axis view using color Doppler imaging demonstrated an antegrade flow from the completely severed main pulmonary artery to both the right and left pulmonary arteries due to BTS, which connected the brachiocephalic artery to the main pulmonary artery (Fig. 1c; Additional file 4: Video Clip 4). The intraoperative clinical course was uneventful. Pre- and postprocedural hemodynamic parameters were as shown in Table 1. The intraventricular blood flow, energy loss, and kinetic energy of the left ventricular outflow tract were assessed using a vector flow mapping software (Hitachi, Tokyo, Japan) in the midesophageal long-axis view by TEE during the surgery (Figs. 2A, A’, B, B’ and 3; Additional files 5, 6, 7 and 8: Video Clips 5–8). Intraventricular energy loss and kinetic energy of the left ventricular outflow tract were higher in the postoperative phase than in the preoperative phase (Fig. 3). The mean energy loss and kinetic energy increased from 29.4 mW/m to 41.9 mW/m and 35.6 mW/m to 83.8 mW/m, respectively. The patient’s pulmonary blood flow progressively worsened with age; therefore, he underwent BCPS and BTS division for second palliation at the age of 11 months. After the procedure, a midesophageal ascending aortic short-axis view by intraoperative TEE revealed a retrograde flow from the right pulmonary artery to the left pulmonary artery due to BCPS, which connected the superior vena cava with the right pulmonary artery (Fig. 1d; Additional file 9: Video Clip 9). Pre- and postprocedural hemodynamic parameters were as shown in Table 1. Vector flow mapping analysis was performed again for this second palliation (Figs. 2C, C’, D, D’ and 3; Additional files 10, 11, 12 and 13: Video Clips 10–13). Intraventricular energy loss and kinetic energy of the left ventricular outflow tract were lower in the postoperative phase than in the preoperative phase (Fig. 3). The mean energy loss and kinetic energy decreased from 38.3 mW/m to 30.7 mW/m and 127.4 mW/m to 62.0 mW/m, respectively.

**Table 1:** Intraoperative hemodynamic parameters

	pre BTS	post BTS	pre BCPS	post BCPS
HR (bpm)	137	156	131	125
BP (mmHg)	65/43	74/37	75/42	72/45
SpO_2_ (%)	84	88	82	94
FiO_2_	0.73	0.47	0.33	1.0
CVP (mmHg)	11	15	13	16

*BTS* Blalock-Taussig shunt, *BCPS* bidirectional cavopulmonary shunt, *HR* heart rate,

*BP* blood pressure, *CVP* central venous pressure

## Discussion

Vector flow mapping is a novel technology that enables the evaluation of intracardiac flow and calculation of energy loss and kinetic energy [3]. This technology uses both color Doppler and speckle tracking images applied to continuity equation from the left and right boundaries. The calculated velocity vectors are integrated according to a weight function [4, 5]. Intracardiac energy loss can be calculated using the following equation [5]:

$$ \boldsymbol{Energy}\boldsymbol{Loss}=\int \mu \left\{2{\left(\frac{\partial u}{\partial x}\right)}^2+2{\left(\frac{\partial v}{\partial y}\right)}^2+{\left(\frac{\partial u}{\partial y}+\frac{\partial v}{\partial x}\right)}^2\right\} dA, $$

where *μ* is the viscosity of blood, *u* and *v* are velocity components along the Cartesian axes (*x* and *y*), and *A* is the area of the unit of the grid.

As the equation indicates, energy loss is the total of squared differences between neighboring velocity vectors which were calculated by vector flow mapping method. It increases with a change in the size and direction of the velocity vectors. For example, energy loss is likely to increase due to turbulent flow caused by factors such as aortic stenosis or an unnatural intracardiac vortex due to surgery [6–8].

The kinetic energy of the left ventricular outflow tract can be calculated from the following equation:

$$ \boldsymbol{KE}=\int \frac{1}{2}\rho {v}^2\times vdL, $$

where *ρ* is the density of blood (1060 kg/m^3^), *v* is the velocity vector of the blood flow, and *dL* is an increment of the cross-sectional line.

Energy loss is considered to be related to prognosis [9]. It is important to take into account the changes in energy loss and kinetic energy postoperatively because both these parameters show an increase in the hyperdynamic state [6].

Staged palliative surgery markedly shifts the balance of volume load on a single ventricle and pulmonary vascular bed. After BTS, a single ventricle serves an important role in both systemic and pulmonary circulations; this ventricle becomes hyperdynamic and the volume load increases as the hemodynamic parameters indicate in Table 1. Energy loss and kinetic energy increase due to the hyperdynamic state. Conversely, after BCPS, the single ventricle is not involved in pulmonary circulation; it becomes hypodynamic and the volume load decreases as the hemodynamic parameters indicate in Table 1. Energy loss and kinetic energy decrease due to the hypodynamic state [10, 11]. These volume loads are difficult to detect using classic hemodynamic parameters. However, we could detect these volume loads using vector flow mapping in terms of energetic performance. After BTS, the increase in kinetic energy (35.6 mW/m to 83.8 mW/m) exceeded the increase in energy loss (29.4 mW/m to 41.9 mW/m) because the single ventricle was additionally involved in pulmonary circulation. Conversely, after BCPS, the single ventricle became hypodynamic, which decreased both energy loss and kinetic energy. The decrease in kinetic energy (127.4 mW/m to 62.0 mW/m) exceeded the decrease in energy loss (38.3 mW/m to 30.7 mW/m) due to the release from pulmonary circulation. Although energy loss is wasted energy, volume loading condition could be estimated by energy loss combined with kinetic energy.

## Conclusions

In the present case, after BTS, the single ventricle became hyperdynamic, which increased both energy loss and kinetic energy. Conversely, after BCPS, the single ventricle became hypodynamic, which decreased both energy loss and kinetic energy. Thus, we demonstrated the change in energetic performance after palliative surgeries.

## Additional files

Additional file 1: Video Clip 1. Midesophageal four-chamber view demonstrating tricuspid atresia, a hypoplastic right ventricle, and VSD before BTS. (MOV 2168 kb)

Additional file 2: Video Clip 2. Midesophageal four-chamber view using color Doppler imaging demonstrating a left to right shunt flow via VSD before BTS. (MOV 2271 kb)

Additional file 3: Video Clip 3. Midesophageal right ventricle inflow–outflow view using color Doppler imaging demonstrating pulmonary stenosis that caused dissipative flow in the main pulmonary artery before BTS. (MOV 2226 kb)

Additional file 4: Video Clip 4. Midesophageal ascending aortic short-axis view using color Doppler imaging demonstrating a continuous antegrade flow from the completely severed main pulmonary artery to both right and left pulmonary arteries due to BTS, which connected the brachiocepharic artery to the main pulmonary artery. (MOV 1858 kb)

Additional file 5: Video Clip 5. Vector flow mapping of midesophageal long-axis view before BTS. (MOV 954 kb)

Additional file 6: Video Clip 6. Vector flow mapping of midesophageal long-axis view after BTS. (MOV 805 kb)

Additional file 7: Video Clip 7. Energy loss in midesophageal long-axis view before BTS. (MOV 917 kb)

Additional file 8: Video Clip 8. Energy loss in midesophageal long-axis view after BTS. (MOV 772 kb)

Additional file 9: Video Clip 9. Midesophageal short-axis view of the ascending aortic short-axis view showing continuous retrograde flow from the right pulmonary artery to the left pulmonary artery due to BCPS which connected the superior vena cava to the right pulmonary artery. (MOV 2122 kb)

Additional file 10: Video Clip 10. Vector flow mapping in midesophageal long-axis view before BCPS. (MOV 925 kb)

Additional file 11: Video Clip 11. Vector flow mapping in midesophageal long-axis view after BCPS. (MOV 956 kb)

Additional file 12: Video Clip 12. Energy loss in midesophageal long-axis view before BCPS. (MOV 899 kb)

Additional file 13: Video Clip 13. Energy loss in midesophageal long-axis view after BCPS. (MOV 932 kb)

## Abbreviations

BCPS: Bidirectional cavopulmonary shunt
BTS: Blalock–Taussig shunt
PDA: Patent ductus arteriosus
TEE: Transesophageal echocardiography
VSD: Ventricular septal defect
